# Serial reversal learning in nectar-feeding bats

**DOI:** 10.1007/s10071-024-01836-y

**Published:** 2024-03-07

**Authors:** Shambhavi Chidambaram, Sabine Wintergerst, Alex Kacelnik, Vladislav Nachev, York Winter

**Affiliations:** 1 Institute of Biology, Humboldt University, Philippstraße 13, 10115 Berlin, Germany; 2 Berlin School of Mind and Brain, Humboldt University, Berlin, Germany; 3 Fairchild Tropical Botanic Garden, Miami, USA; 4 Department of Biology and Pembroke College, University of Oxford, Oxford, UK; 5 Present Address: QUEST Center for Responsible Research, Berlin Institute of Health at Charité–Universitätsmedizin, Charitéplatz 1, 10117 Berlin, Germany

**Keywords:** Serial reversal learning, Bats, *Glossophaga commissarisi*, Behavioral flexibility, Foraging, ‘Win-Stay-Lose-Shift’

## Abstract

We explored the behavioral flexibility of Commissaris’s long-tongued bats through a spatial serial reversal foraging task. Bats kept in captivity for short periods were trained to obtain nectar rewards from two artificial flowers. At any given time, only one of the flowers provided rewards and these reward contingencies reversed in successive blocks of 50 flower visits. All bats detected and responded to reversals by making most of their visits to the currently active flower. As the bats experienced repeated reversals, their preference re-adjusted faster. Although the flower state reversals were theoretically predictable, we did not detect anticipatory behavior, that is, frequency of visits to the alternative flower did not increase within each block as the programmed reversal approached. The net balance of these changes was a progressive improvement in performance in terms of the total proportion of visits allocated to the active flower. The results are compatible with, but do not depend on, the bats displaying an ability to ‘learn to learn’ and show that the dynamics of allocation of effort between food sources can change flexibly according to circumstances.

**Supplementary Information:**

The online version contains supplementary material available at 10.1007/s10071-024-01836-y.

## Introduction

Many animals face frequent and unpredictable changes in the relative profitability of food sources. This is the case for animals that forage for nectar and/or pollen in flowering plants. Though flowers are stationary, changes in their profitability occur at many time scales. Many plants bloom seasonally, and single flowers on plants themselves wither and die every day or every few days. Furthermore, flowers are depleted and replenished at various timescales by the interplay of foragers and nectar production, altering their profitability as food resources with the passage of time.

Behavioral flexibility helps to cope with such changes. The word ‘flexibility’ has been used to mean many different things in the animal behavior literature (often inconsistently—for a discussion, see Audet and Lefebvre 2017), and one of its manifestations relates to the concept of elasticity: behavioral patterns that can be repeatedly and readily reversed (Bond et al. 2007). An experimental protocol that has been widely used to test for and demonstrate this sort of flexibility is reversal learning (Izquierdo et al. 2017).

In a reversal learning task, an animal first learns about multiple stimulus–reward pairings, such as two or more spatial locations that can be visited to potentially obtain rewards. In the initial phase, subjects typically bias their behavior toward the richer location. When contingencies are stable and/or only one of the locations offers rewards, the reward-maximizing strategy is to allocate all behavior to that one. However, if and when the contingencies in the available sources vary with time, behavioral allocation is expected to, and typically is, less extreme, facilitating the detection of other opportunities. The temporal course of such adjustments is an informative index of the difference in flexibility, such as between species (Bond et al. 2007), between sexes (Lois-Milevicich et al. 2021), and between temporal or experienced contingencies (Santos et al. 2021; Smith et al. 2018). Here, we use a reversal protocol to explore flexibility in learning dynamics and how it affects the foraging efficiency of nectar-feeding bats.

In situations where there are two sources of rewards of which at any given time only one is active, but which one it is switches frequently, foraging yield is positively related to behavioral allocation to the currently active source and to the speed of detection of reversals. In these scenarios, there is a trade-off between these two factors, because greater commitment to one source reduces the information available about the state of the other. When reversals are between two continuous reinforcement reward schedules (i.e., when any active source yields food on every visit), the theoretical strategy “Win-Stay, Lose-Shift” (WSLS) is almost perfectly maximizing, since it takes only one unrewarded visit to detect each reversal (minor deviations not being of interest here). However, WSLS has not been found to accurately describe the behavior of experimental animals (Santos et al. 2021; Smith et al. 2018). Instead, typically, behavior is better described by combined sensitivity to schedule properties that, although programmed deterministically, are evidently not perceived as such by the subjects. In some cases, the mechanism by which the subject allocates choices is expressed in continuous (rather than discrete) changes in behavior. For instance, in the mid-session reversal protocol, reversals are programmed to occur after a fixed number of trials, which usually corresponds with a somewhat predictable point in time. Using pigeons as subjects, Smith et al. (2018) found that as a reversal approached, in trials where subjects faced only one option rather than a binary choice, pigeons showed a smoothly increasing latency to respond to the currently rewarding option, and a decreasing latency to respond to the soon-to-be-rewarding option. In intermingled choice trials, there were both anticipatory and perseverative choices of the currently non-rewarding option. Thus, the independent smooth variation in latencies in single option trials correlated, probably causally, to the gradual and probabilistic distribution of choices in two-option trials, a regularity that describes well behavior in other protocols (Kacelnik et al. 2011). Notice that we avoid labeling all choices of the currently non-rewarding options as ‘errors’ as these unrewarded attempts may be the outcome of an efficient strategy given the information available to the subjects.

In serial reversal learning procedures, where reward contingencies reverse repeatedly, subjects can use their experience to adjust the dynamics of behavior allocation so as to improve reward yield. In other words, subjects could show the second-order learning, or learning to learn, similar to observations in ‘learning set’ protocols (Harlow 1949). This could involve learning that the sudden absence of reward signals reliably that reward contingencies have reversed.

We carried out a serial reversal learning task with Commissaris’s long-tongued bats (*Glossophaga commissarisi*), which primarily feed on flower nectar (Tschapka 2004). They may often experience unstable opportunities in their natural environment, because a flower may remain rewarding for multiple visits before it is depleted by self or a competitor, or withers and dies. When a given flower becomes inactive, the memory of previously rewarding flowers that may have replenished is likely to drive visits to nearby alternatives. In our experiment, bats were offered two potentially rewarding options, identified by their spatial location. Glossophagine bats have well-developed spatial memory (Stich 2004—chapter 2; Stich 2004—chapter 3; Thiele and Winter 2005; Winter et al. 2005), so no other cue was necessary. At the start of each night, one of the options was active, yielding nectar every time it was visited (the ‘S + ’ option), while the alternative was inactive (the ‘S-’ option). After a fixed number of visits, the reward contingencies reversed without any additional cue: the rewarding option became inactive (S +--> S-) and the previously unrewarding option became active (S---> S +). This reversal happened five times in a night.

In the wild, when new flowers become available, bats can quickly learn the locations associated with high reward, a form of first-order learning (Nachev et al. 2017; Nachev and Winter 2012; Stich 2004—chapter 2; Stich 2004—chapter 3; Tölch and Winter 2007; Winter et al. 2005). It has also been shown that bats learn a change in spatial location much faster than when flowers change in echo-acoustic properties (Thiele and Winter 2005). Frequent changes in flower properties may involve second-order learning, in which bats’ behavioral trajectory of biasing toward present relative profitability reflects not just present reward but also the dynamics of previously experienced opportunity fluctuations. The aim of our present experiment was to test if bats show flexibility in their adjustment to repeated reversal of contingencies, so that experience with changes translated into improvements in foraging efficiency.

Since in our protocol, there is a simple, near-optimal theoretical strategy, in the form of win-stay lose-shift, we were also interested in finding out whether the bats progressively approached this strategy by learning to reallocate behavior more abruptly after just one unrewarded visit.

## Methods

### Study site and subjects

The experiment took place at the Organization for Tropical Studies (O.T.S/O.E.T) La Selva Biological Field Station, Province Heredia, Costa Rica in June–July 2017. *Glossophaga commissarisi* bats were captured and retained in a flight cage throughout the experiment. The bats were attracted to a particular location in the forest using chicken-feeders filled with sugar water (see *Reward*) as bait. The feeders had cotton swabs soaked in dimethyl disulphide on them, a chemical odor attractant produced by many bat-pollinated flowers (von Helversen et al. 2000) and then caught in mist-nets. The bats were sexed on capture, pregnant or lactating females excluded, and housed in two outdoor, meshed flight cages (4 × 6 m) under ambient light conditions. All individuals were weighed and marked with radio frequency identification (RFID) tags placed as collars around their necks.

A total of 16 bats participated in the main experiment, and the first stage of the experiment began on the night the bats entered the cages. A group of four experimental bats of the same sex were placed in a flight cage together. Two such groups were run in parallel, one in each flight cage, so the data were collected simultaneously. At the end of the experiment, the RFID collars were removed, and the bats were released back into the wild. All the data collection was completely automated. Two of the bats did not drink a sufficient amount of sugar water to meet minimum energy requirements. These two bats were released before the end of the experiment and not replaced, and data from these two individuals were not analyzed. Thus, 14 bats (7 males and 7 females) completed the experiment. Permission for this research was granted by Sistema Nacional de Areas de Conservación (SINAC) at the Ministerio de Ambiente y Energía (MINAE), Costa Rica.

### Experimental setup

### Reward

The rewards consumed by the bats during the experiment were also their main source of food. We used a 17% by weight solution of sugar dissolved in water, hereafter referred to as ‘nectar.’ The sugar consisted of a 2:1 mass mixture of fructose and glucose, an approximation of the floral nectar composition of chiropterophilous plants (Baker et al. 1998). Every night, the bats were also given supplemental food in the flight cage in a bowl accessible to all: per bat this was 0.25 mL of honey and 0.3 g of milk powder (Nido 1 + , Nestle, Switzerland) dissolved in 1 mL of water. In addition, a bowl of locally sourced bee pollen was placed in each cage every night.

### Flower and feeder setup

**Fig. 1:**
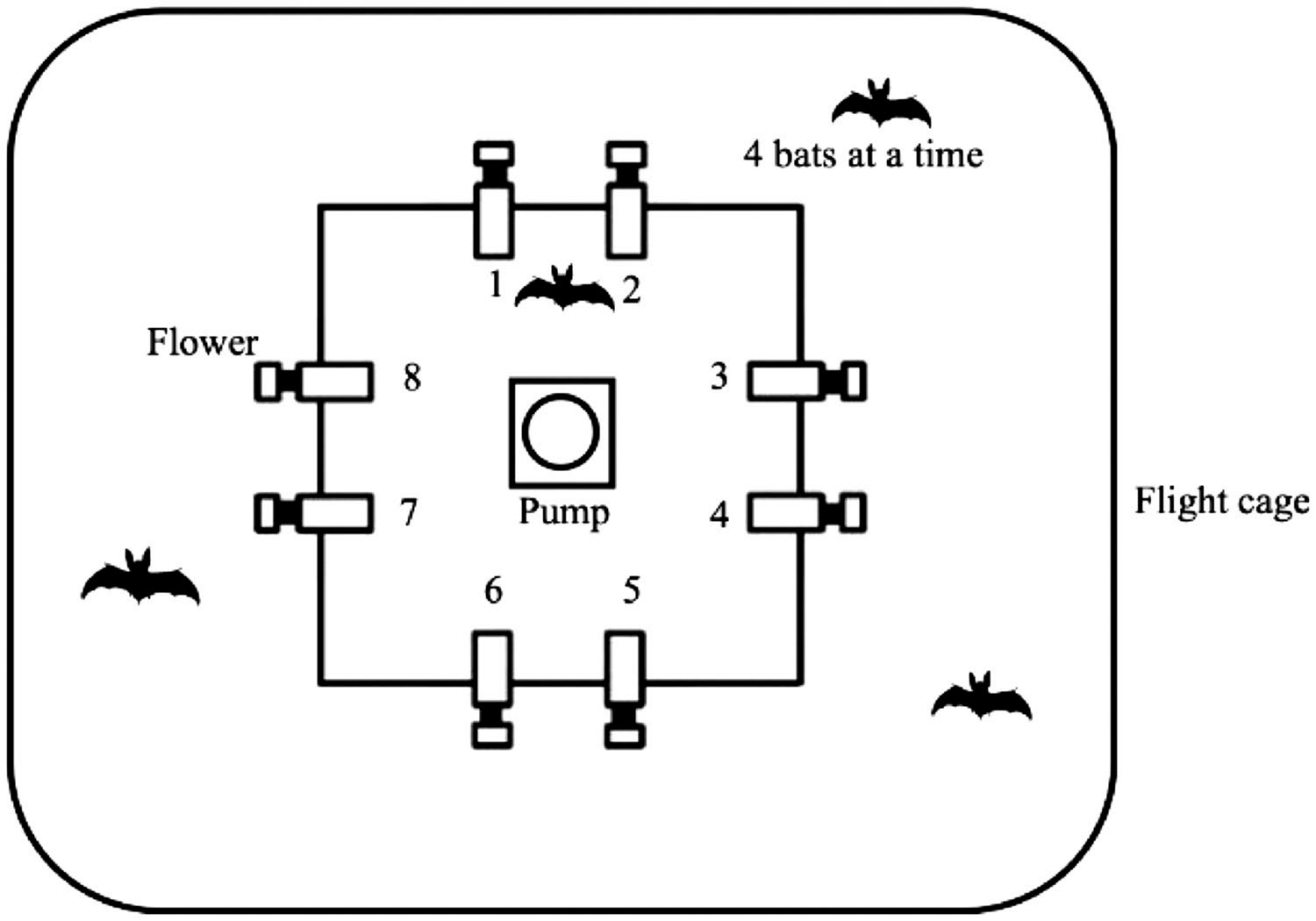
Schematic of the cage and flower setup (not drawn to scale). Dimensions of the flight cage were 4 × 6 m. The square holding the artificial flowers measured 2 × 2 m and was 1.5 m above ground. Neighboring flowers were 40 cm apart

Each flight cage had a square frame in the center (2 × 2 m), fixed 1.5 m above the ground. Eight reward-dispensing devices—hereafter referred to as ‘flowers’—were fixed two on each side of the square (Fig. 1) with a distance of 40 cm between adjacent flowers. At this separation, bats can fully discriminate neighboring spatial locations (Thiele and Winter 2005; Tölch and Winter 2007). Each flower had the following parts: a circular RFID antenna mounted at the end of a plastic cylinder that constituted the artificial flower; an infrared photo gate; and an electronic pinch valve through which a silicon tube was placed and fixed to the base of the flower (see Thiele 2006 and Winter and Stich 2005 for details of the equipment).

A stepper-motor syringe pump was placed in the center of the square in each cage with a 25 mL Hamilton glass syringe (Sigma-Aldrich, Germany). All bats received the same volume of reward on each visit: 40 μL of nectar. The syringe was connected to the tubing system of the flowers through five pinch valves (Nachev et al. 2012). The pinch valves controlled the flow of liquid from the pump to the flower system and from a reservoir of liquid to the pump. The reservoir (500 mL bottle, Roth, Germany) was filled with fresh nectar every day.

Every day, an automated routine was started at around 10:00. The system was emptied of remaining nectar and rinsed with plain water. The system was then filled with water and kept this way until 15:00, when it was filled with fresh nectar. Twice a week, the system was filled with 70% ethanol for an hour to prevent microbial growth and then rinsed with water.

When a tagged bat approached a flower, its individual number was read. If the bat then poked its nose into the flower and interrupted the light beam, this was recorded, and if it was an assigned flower and currently active, a reward was released. The flowers were programmed, such that each bat could trigger rewards at only two unique flowers out of the array of eight. Upon a nose poke, the pinch valve opened the tubing connection to the nectar pump, and the pump dispensed nectar to the base of the flower. The bat could hover in front of the flower and lick up the nectar. The flowers and the pump were connected to a Windows PC, which ran the experimental programs, collected the data, and also ran the routine program used to automatically flush, clean, and fill the pump and tubing system (PhenoSoft Control, PhenoSys, Germany).

### Experimental procedure

Out of the array of eight flowers, each bat was assigned two adjacent flowers on the same side of the square frame, programmed to reward only one of the bats in the cage. After the system was filled with fresh nectar at 1500 h, the program was launched at approximately 1700 h and left running for data collection till the next morning. Thus, the bats could begin visiting the baited flowers whenever they chose, which was at nightfall, at approximately 1800 h every night. During the main experiment, each bat could make a maximum of 300 rewarded visits a night, after which both flowers would cease to offer rewards. However, the bats had consumed enough nectar to be satiated before reaching this limit, and there were very few visits after the flowers ceased to offer rewards.

During the course of the night, when the syringe had been emptied, the pump re-filled automatically. This happened only once every night and it took 4.5 min (SD =  ± 0.18) for the cage 1 pump, and 2.4 min (SD =  ± 0.04) for the cage 2 pump.

About 1% (SD =  ± 0.74) of all visits made by the bats were wrongly unrewarded, meaning that a bat did not receive a reward even when the visit was made to a flower that was supposed to be rewarding at the time. This happened either during the pump refill times or when the pump was moving to reward a visit made by another bat that happened almost at the same time. Such events did not count toward the total of 300.

### Experimental design

The experiment proceeded through the following stages.

### Training

On the night, the naïve bats were captured and placed into the flight cages, they could receive a reward from any of the flowers whenever they visited them throughout the night. To facilitate a fast learning of our artificial flowers as locations of reward, a small cotton pad soaked in dimethyl disulphide was placed on each flower to encourage the bats to explore the flower heads for nectar food, interrupt the photo gate, and trigger a nectar reward. By the end of the night, all the bats had found the flowers and learned to trigger rewards.

The next stage of training involved assigning each bat to two of the eight flowers in the array. For an individual animal, only the two flowers assigned to it would elicit rewards from this stage of training until the end of the experiment. This stage was similar to the previous one, except that the bats could only collect nectar from their two assigned flowers. The chemical attractant was not used.

The final stage of training was forced alternation. The bats received a reward at one of the two flowers for one trial, and then could only receive reward at the other flower for the next trial. If the bat repeatedly visited the same flower, the other flower would remain rewarding until it was visited. The purpose of this training stage was: to pre-expose the bats to the location of the two potentially rewarding flowers; to counteract their natural bias to be faithful to a previously rewarding site; and to neutralize pre-existing side preferences. This pre-training may have increased their tendency to switch with respect to a naïve individual, thus decreasing persistence after reward extinction. Because the hypothesized effect is in the opposite direction (i.e., we expect and measure an increasing readiness to switch after a flower ceases to offer nectar), alternating pre-training is unlikely to have enhanced our results.

### Serial reversal learning task

In the serial reversal learning stage, for any given bat at any given time, one of its two flowers gave a 40 μL nectar reward, and the other was unrewarding. After a bat had made 50 visits in total to its assigned two flowers, a reversal occurred: the previously rewarding flower became non-rewarding and vice versa. Only visits to the two flowers assigned to each bat counted toward its visit tally, and the distribution of visits between these two flowers did not have any effect. Each set of 50 visits was termed a ‘block.’ Each bat had six blocks and five reversals per night, unless it entirely ceased visiting the flowers earlier. This was repeated for three consecutive nights, and the same flower started the sequence every night. However, since bats already during the first night seemed to have reached their asymptote of quickly adjusting their choices after a reversal had occurred, for this study, we focus on the statistical analysis of the five reversals experienced the first night.

### Data analysis

The raw data collected during this study were the computer-logged events of feeder visits. Each event included the time stamp, animal ID, photo gate interruption duration, and the volume of nectar dispensed: either 40 μL or 0 μL. The bats made occasional visits and approaches to the flowers that were not assigned to them. However, these visits were infrequent: they made up less than 10% of the overall number of visits and were not considered for the analysis (see *Supplementary Material* for details). For the analysis, blocks were further divided into five bins of ten visits, to examine the bats’ behavior within each block. R (version 3.6.3, R Development Core Team 2020) was used for all statistical analyses and creation of plots.

All the statistical models were fitted in a Bayesian framework using Hamiltonian Monte Carlo in the R package brms (Bürkner 2017) which is a front end for rstan (Carpenter et al. 2017). Generalized linear mixed models were used for the analyses (see *Supplementary Material* for the technical details of the model fitting). Unless mentioned otherwise, we report here the mean as a measure of central tendency and the 89% quantile-based credible intervals for the intercept and slope coefficients (89% boundaries are the default for reporting credible intervals—McElreath 2020). To aid in the interpretation of the model parameters, we also present plots of the conditional effects of some of the predictor variables.

**Fig. 2:**
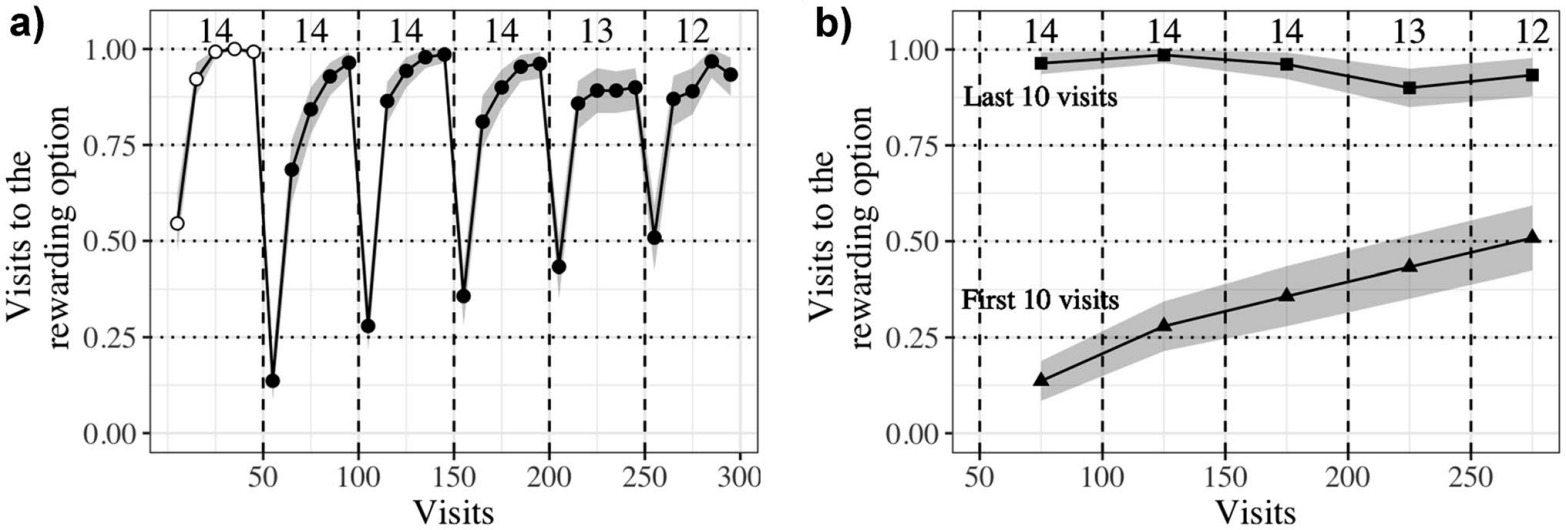
Visits to the rewarding one of two options. Shading shows 95% confidence intervals, and numbers indicate the total number of bats that participated in a block. Data are average proportions for bins of ten visits averaged over all the individuals that made visits in each bin. Vertical dashed lines show reversals **a** Data indicated by white circles in the first block were before the bats had experienced any reversals; black circles after the experience of a reversal. **b** Data indicated by triangles are proportions of rewarded visits in the first bin of ten visits in a block; by squares, in the last bin of ten visits in a block

Visual inspection of the whole data set showed a qualitative difference between the first and later nights, implying that any second-order learning effect already reached asymptotic stability by the 5th reversal at the end of the first night (see Fig. 2) To avoid masking first-night acquisition effects by swamping them with post-asymptotic (overtraining) data, we focus on the first night in our statistical analysis. The data from and analysis of the second and third nights are presented in full in the supplementary material.

The first reversal was experienced at the end of the first block of the first night, namely by the 51st flower visit for each bat. Hence, this is where we started the analysis to seek for evidence of second-order learning. We used the proportion of visits to each of the two flowers in each 10-visit bin as response variable and fitted a generalized linear mixed model (GLMM) using bin and reversal number and their interaction, as explanatory variables. The proportion of visits to the rewarding flower was given by the number of visits to the S + divided by 10, which was the total of visits made to both flowers in each bin. This was denoted as the Prop_rew_. Confidence intervals were calculated by non-parametric bootstrapping using the Hmisc package (Harrell 2021).

## Results

During the first experimental night and after the first 20 visits, bats made more than 95% of their visits to the rewarding flower.

Figure 2a shows the temporal adjustment of visit allocations between the assigned flowers, highlighting both the effect of experience within a block and between blocks (i.e., reversals). Within each block, allocation to the rewarding flower increased with successive bins, progressing toward an asymptotic proportion. After each reversal, persistence to the previously rewarding source was brief—though longer than the single unrewarded attempt expected from win-stay lose-shift. Thus, within-block behavioral allocation changed as the bats’ responses quickly evolved toward a new asymptotic distribution with a bias toward the currently active source of nectar.

At the start of the experiment, in the first bin of ten visits when the bats had not experienced the reward contingencies, Prop_rew_ (the proportion of visits to the rewarding option) averaged across individuals was at chance level: 54.5% [95% CI 46.8, 62.3], about half of the ten visits. Within the next ten visits, however, mean Prop_rew_ increased to 92.1% [95% CI 87.1, 96.4] and by the last bin of this first block was almost completely directed at the rewarding flower, at 99.3% [95% CI 97.9, 100]. Reallocation after the first reversal was fast, as Prop_rew_ already reached 13.6% [95% CI 8.4, 18.8] within the first ten visits, reaching a Prop_rew_ of 96.4% [95% CI 92.9, 99.3] by the last bin of this block (Fig. 2b). There was a trend toward faster re-allocation as experience of reversals increased (Fig. 2b).

**Fig. 3:**
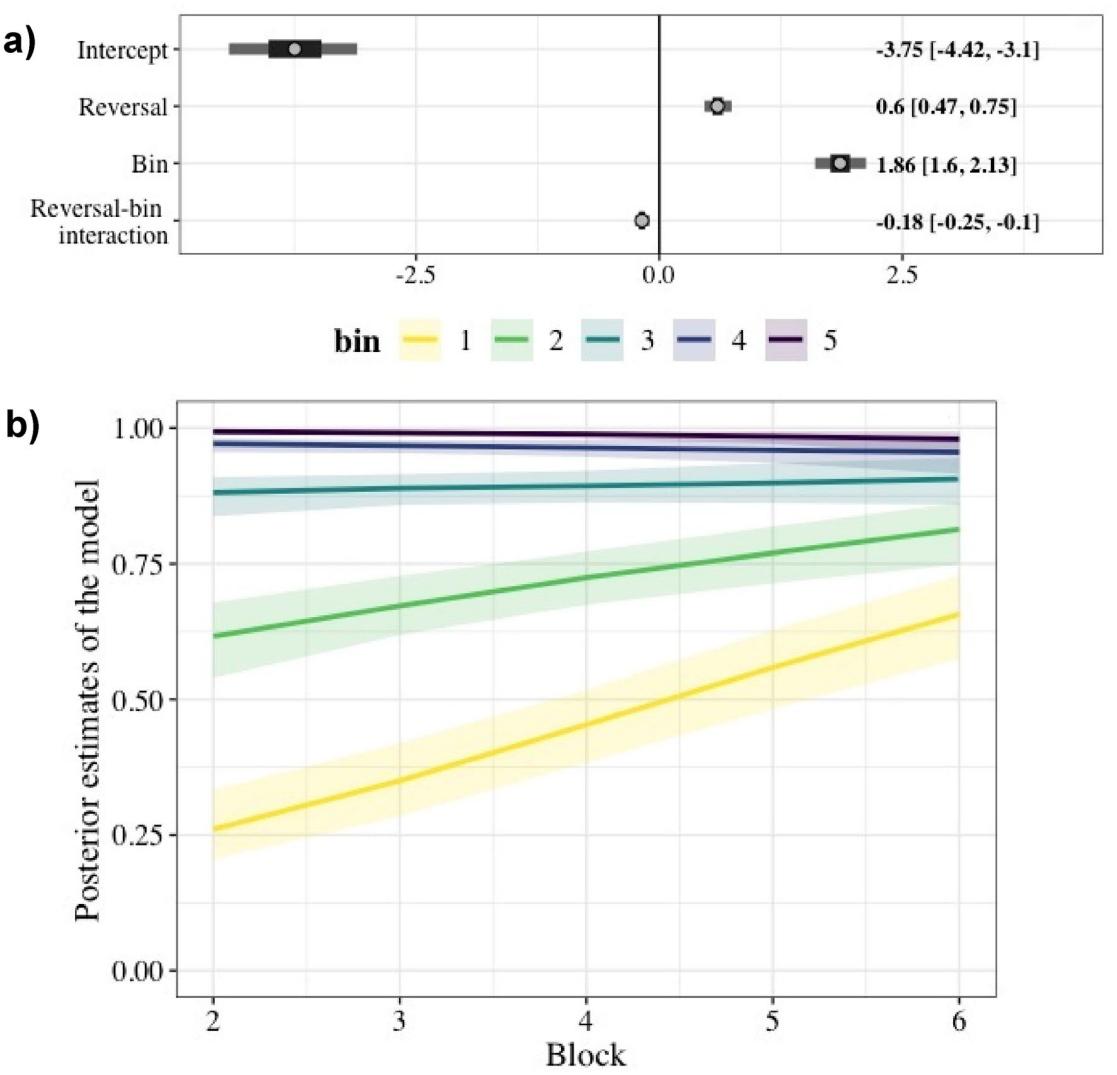
**a** Forest plot of the regression coefficients from the model of the effect of reversal and ten-visit bin on the visits to the rewarding flower. Data are means and their 50% and 89% credible intervals of the posterior distributions of the slope coefficients, with their values given on the right. **b** Effect of number of blocks on the proportion of correct choices for the five consecutive ten-visit bins (colors yellow to purple) within a block. Lines are the conditional effects plot from the model of the effect of reversal and ten-visit bin on the visits to the rewarding flower showing the effect of reversal and bin, sampling from the posterior distribution

The most salient signal for second-order behavioral change was the decline in perseverative visits to the previously rewarding option after consecutive reversals. The proportion of visits to the presently rewarding flower in the first bin of ten visits after a reversal increased more than in any other bin as the bats accumulated experience. This is observable in the raw data (Fig. 2b; see also Figure S3 in the *Supplementary Material*) and held up by the statistical analysis (Fig. 3).

Statistically, Prop_rew_ in the first bin of each block increased significantly as the bats experienced successive reversals. This reversal-dependent or second-order effect was present in the second bin of ten visits (visits 11–20) as well, but by the third bin, the reversal effect was not statistically detectable (Fig. 3b). The mechanism for the acceleration of switching could depend on the bats forming an expectation that things change after about 50 visits, leading to an increase in the proportion of win-shift (“proactive sampling”) responses toward the end of each block. An alternative, not mutually exclusive, mechanism could be an increase in lose-shift probability, which would be evidenced by the dynamics at the beginning of each block, after reversals. There is no strong evidence of the former (but see the trend to lower asymptotes in Fig. 2), but there is reliable statistical evidence for the latter (see Fig. 3). On balance, one may expect both factors to play some role.

When the bats had their very first experience of a reversal, the proportion of rewarded visits dropped to the lowest level of the whole experiment. Therefore, we asked whether the first reversal just by itself was responsible for the significance of the factor *number of reversal* on Prop_rew_ in our statistical model of the data set. To investigate this, we removed these data and repeated the analysis. We found that the effect of the reversals on the Prop_rew_ persisted, even without the effect of the first reversal (see *Supplementary Material*, Figure S4).

## Discussion

We studied temporarily captive wild nectar-feeding bats in a spatial serial reversal learning task with two food sources that repeatedly alternated their rewarding properties, seeking evidence for behavioral flexibility in the mechanisms by which bats adjust to dynamically changing foraging environments. From a functional perspective, we sought to characterize the consequences of such adjustments in terms of payoffs. We found that as bats experienced more reversals, they reduced the number of perseverative failures after each reversal. In addition, there was a trend to decrease their asymptotic commitment to the presently better source. On balance, these two effects resulted in an increase in the total proportion of visits made to active flowers, leading to better yields.

In this protocol, the theoretical Win-Stay, Lose-Shift strategy would yield virtually maximal payoffs (losing only one reward per reversal). The bats did not follow WSLS, but they did increase the proportion of shifts (i.e., visits to the alternative flower) after a loss (an unrewarded visit), thus reducing the deviation from WSLS. This ‘speed of switching’ increased within five experiences of reward reversal, in their first experimental night. Further experience, in two follow-up nights with five reversals each, did not yield further detectable changes (see *Supplementary Material*), indicating a protocol-dependent ceiling in performance.

The probability of returning after a reward (win-stay) was high from the start, and an increase as a function of reversals would have been harder to detect precisely, because it was already very high from the first block onward. It would be misleading to label deviations from WSLS as anticipatory or perseverative ‘errors’: Although in the experimental protocol, contingencies changed precisely every 50 visits, the bats had no reason to be perfectly tuned to this. Changes in natural flowers are likely to be temporally noisy, and a probabilistic approximation to the WSLS policy may provide a good balance between sampling and exploitation.

We were interested in demonstrating a learning property: the ability to adjust to temporal properties of food sources. The mechanisms revealed in our experiments are bound to be present in nature. However, to form a picture of the ecological relevance of our results, it is pertinent to highlight some differences between our protocol and natural situations. First, our bats were not deprived, as we used a large reward size (40 μL) and supplementary food was also available. Levels of deprivation are bound to vary widely in the wild, and this may have complex effects on learning, decision-making, and the balance between exploration and exploitation. It has been shown for example that many animals, including a species of bat, show state-dependent learning, or evaluate rewards more highly when they are obtained under a state of deprivation (Hemingway et al. 2020; Pompilio et al. 2006; Pompilio and Kacelnik 2005). Second, we offered two flowers with deterministic properties. In nature, the set of potential food sources is open and from a forager’s perspective varies stochastically due to fluctuations imposed, among other factors, by competition. These caveats are ubiquitous: laboratory experiments expose behavioral mechanisms, but do not substitute empirical research in the ecological circumstances to which behavior is adapted.

That said, it is also noteworthy that though most flowers in nature are emptied in a single visit, there are certain plants such as species of *Agave* or *Vriesea*, that may hold large amounts of nectar. If undetected for a long time, such flowers require multiple hovering visits to deplete—in other words, they are “jackpot” rewards. Nevertheless, such flowers may also be depleted suddenly due to the presence of competitors for the nectar. Thus, the ability to swiftly inhibit visiting a flower that had been rewarding for multiple visits is likely bats’ natural foraging ecology as nectar-feeding animals.

Bat behavior and learning processes are surely adapted to fit the complexity of their environment and may reflect priors and learning processes that are effective in such scenarios. This general observation does not question the reliability of laboratory findings, but is a reminder of the caution that must be shown in extrapolating to natural circumstances, especially when aspects of behavior are, as sometimes happens, labeled as being ‘errors’ or being ‘suboptimal’.

After the bats experienced no reward at a hitherto-rewarding option for the first time, the proportion of rewarded visits decreased, and never again showed the nearly exclusive preference for the rewarding flower that they displayed at the end of the first block. Bats, like most vertebrate foragers, are known to adjust their choice behavior between different available options according to their history of reinforcement at those options (Nachev and Winter 2012). If it is solely reinforcement history that dictates choice behavior, regardless of temporal structure and dynamics, then as experience of reinforcement accumulates at both flowers over the course of a night, it should become progressively more difficult to discriminate which flower has a richer history. In this case, one would expect that the bats’ behavior would approach random choice as the night goes on, i.e., Prop_rew_ would approach 0.5 and there would be slower modification of behavioral allocation. Even if the animals rely on only a part of their history of reinforcement at an option, and not the whole history, one would expect to see a slower switch to the rewarding option after a reversal, and then an increase in rewarded visits. This is the opposite of what was seen: with more reversal experience, the bats switched to the newly rewarding option after increasingly fewer choices. However, behavioral allocation at the end of each block did show a weak trend to progressively become less extreme as reversal experience accumulated (Fig. 4b, bins 4 and 5), and the weight of cumulative past reinforcement may be a contributing factor in this.

While it is clear, therefore, that the bats’ choice behavior was not dictated by the cumulative reinforcement history at the two options, the readiness to switch to the rewarding option reached a maximum by the end of the first night, after five reversals (see *Supplementary Material*). It is interesting that while appropriate re-allocation became swifter after successive reversals, the theoretical reward-maximizing strategy (Lose-Shift), which would incur only one unrewarded visit per block, was never reached, and both speed of switching and asymptotic commitment to the better option seemed to reach a limit toward the end of the first night. In fact, total commitment to the best option in a set is not to be expected if a forager is adapted to track changes in its environment, a point already made in the early foraging literature (e.g., Smith and Sweatman 1974). Indeed, we note that the bats in our experiment made approximately 10% of their visits every night to the six flowers that were not assigned to them—and were therefore always non-rewarding—out of the array of eight (Figure S1 and S2 in the Supplementary* Material*). This behavior occurred even though the non-assigned flowers were only rewarding on the very first night of training, followed by several nights of being consistently non-rewarding.

What performance on the serial reversal task says about the cognitive mechanisms at work is not completely settled. Cognitive flexibility relies on processes in the brain that permit adaptive change in behavior in response to changes in the internal or external environment, whereas behavioral flexibility is the modifiability of learned behavior (Dhawan et al. 2019). Cognitive flexibility cannot be directly observed; it is inferred to have occurred through behavioral flexibility (Tait et al. 2018), and the reversal learning task has been variously considered as a test of cognitive flexibility (Izquierdo et al. 2017) and behavioral flexibility (Dhawan et al. 2019).

Behavioral flexibility has been found in most species tested in reversal learning tasks with widely varying foraging ecologies. This includes our own study species (Thiele 2006), bumblebees (Chittka 1998; Strang and Sherry 2014), honeybees (Menzel 1969; Mota and Martin 2010), rats (Dhawan et al. 2019; Mackintosh 1965; BvG and El Massioui 2000), pigeons (Williams 1967), rabbits (Orona et al. 1982), corvids (Bond et al. 2007), pigeons (Diekamp et al. 1999), marmosets (Clarke et al. 2008), and even human children (Eimas 1966). It is also present in species adapted to different spatial demands, such as hoarding species of passerines such as black-capped chickadees (Hampton et al. 1998), Clark’s nutcrackers (Lewis and Kamil 2006), and high elevation mountain chickadees (Croston et al. 2017), and in brood parasitic birds that must keep some form of ‘book-keeping’ regarding the state and availability of potential target host nests (Guigueno et al. 2014, 2016; Lois-Milevicich et al. 2021).

The processes and parameters of first- and second-order learning about the location of reward sources, the availability and properties of rewards, and other qualitative and quantitative details must adaptively reflect the biology of each species. However, through these differences, a common picture emerges in which animals display general and remarkable abilities to pick up the relevant environmental affordances at various temporal and spatial scales.

## Supplementary Information

Below is the link to the electronic supplementary material.

Supplementary file1 (PDF 722 KB)

## Data Availability

All data and code are available in the Zenodo repository: 10.5281/zenodo.10430353.
