# Supplementary material for: Serial reversal learning in nectar-feeding bats
Source: Anim Cogn. 2024 Mar 7;27(1):24. doi: 10.1007/s10071-024-01836-y (PMC10920430; doi:10.1007/s10071-024-01836-y)
Supplement: Supplementary file 1 — Supplementary file1 (PDF 722 KB) [file 10071_2024_1836_MOESM1_ESM.pdf]

Serial reversal learning in nectar-feeding bats

Animal Cognition

Shambhavi Chidambaram[1,2] (ORCID: 0000-0002-8560-2304) Sabine Wintergerst[3], Alex Kacelnik[4] (ORCID: 0000-0002-3188-8255), Vladislav Nachev[1,5] (ORCID: 0000-0003-0521-6153), York Winter[1,2*] (ORCID: 0000-0002-7828-1872)

[1] Institute of Biology, Humboldt University, Berlin, Germany

[2] Berlin School of Mind and Brain, Humboldt University, Berlin, Germany

[3] Fairchild Tropical Botanic Garden, Miami, USA

[4]Department of Biology and Pembroke College, University of Oxford, UK

[5]Berlin Institute of Health (BIH) at Charité - BIH QUEST Center for Responsible Research, Berlin, Germany (present affiliation)

[*]**For correspondence:** york.winter@hu-berlin.de

Present Address: Institute of Biology, Humboldt University, Philippstr. 13, 10115 Berlin, Germany

Key words: Serial reversal learning; bats; *Glossophaga commissarisi*; behavioural flexibility; foraging; ‘Win-Stay-Lose-Shift’

Electronic Supplementary Material

Visits and approaches to the unassigned flowers

Only two out of the array of eight flowers were assigned uniquely to each bat but all the flowers were accessible to all the animals. The number of attempts to get a reward from all the flowers, both assigned and not assigned, is shown in Figure S1.

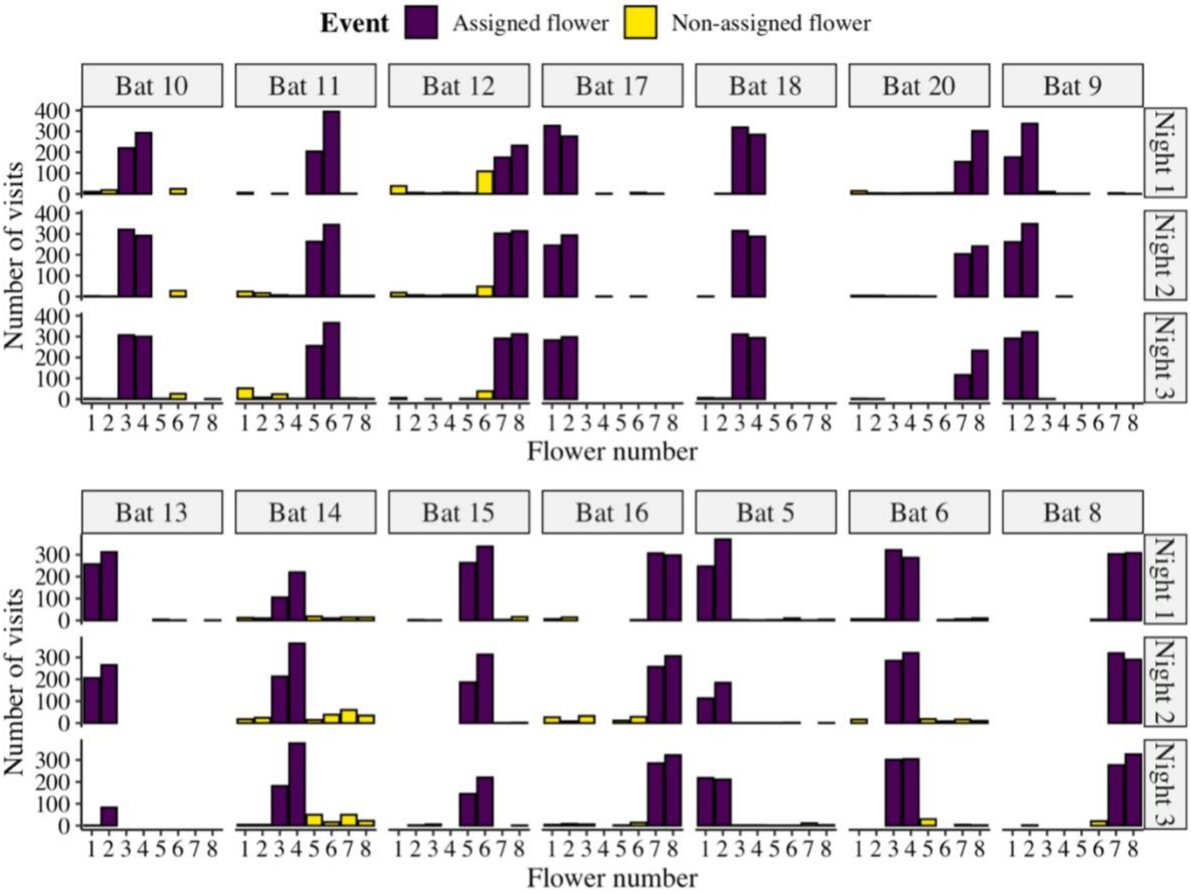

Fig S1 Visits made by the bats to all the flowers, including the ones that were not assigned to them. Purple bars are nose-pokes at the assigned flowers, where the bats attempted to get a reward by breaking the light-barrier. Yellow bars are nose-pokes at the non-assigned flowers

The number of attempts to get a reward at the non-assigned flowers was a small proportion of the overall number of approaches and reward-attempts at the flowers, less than 10% every night on average as Figure S2 shows.

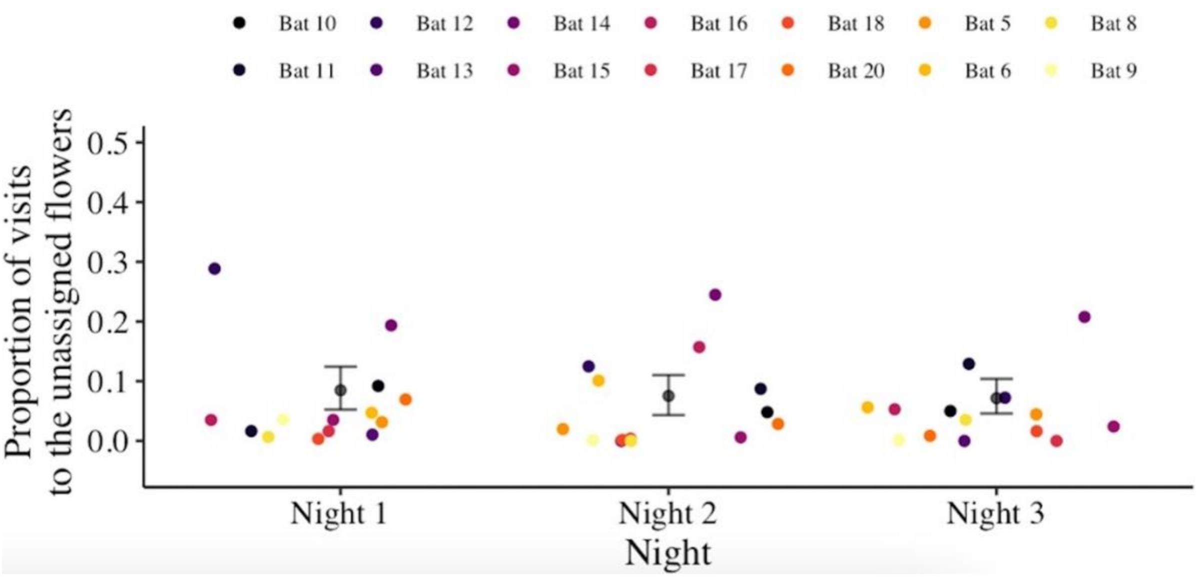

Fig S2 Proportion of visits to the un-assigned flowers out of the total number of visits to flowers. Colored points are data from individual bats. Black points are the mean proportion per night and the error bars are 89% CIs

Details of the statistical analyses

Weakly informative priors were used for the GLMMs in brms. All the models were estimated using 4 chains with a thinning interval of 3, with 1500 warm-up samples and 3000 post-warm-up samples for all the models except the one fitted to the data from the second and third nights, which had 1000 warm-up samples and 3000 post-warm-up samples. The response variable was the proportion of visits to the rewarding option, and a Bernoulli likelihood function was used. For the model of the first experimental night, the reversal number, 10-visit bin within each block, and their interaction were the fixed effects. Slopes and intercepts were allowed to vary for each animal. For the model of the second and third experimental nights, the experimental night, reversal number, 10-visit bin within each block, night-reversal interaction and reversal-bin interaction were the fixed effects. Slopes and intercepts were allowed to vary for each animal.

Visual inspection of the trace plots, the effective sample size, the Gelman-Rubin convergence diagnostic ($\hat{R}$) and the calculation of posterior predictions for the same clusters were all used to assess the fit of the models. In all the models the $\hat{R}$ was equal to 1 for all the chains.

Perseverative visits made by the bats on the first experimental night

The number of visits made after a reversal to the previously rewarding flower, i.e., the number of perseverative visits, declined with each successive experience of a reversal on the first experimental night. The raw data from the individual bats on the first night are depicted in Figure S3 showing this trend.

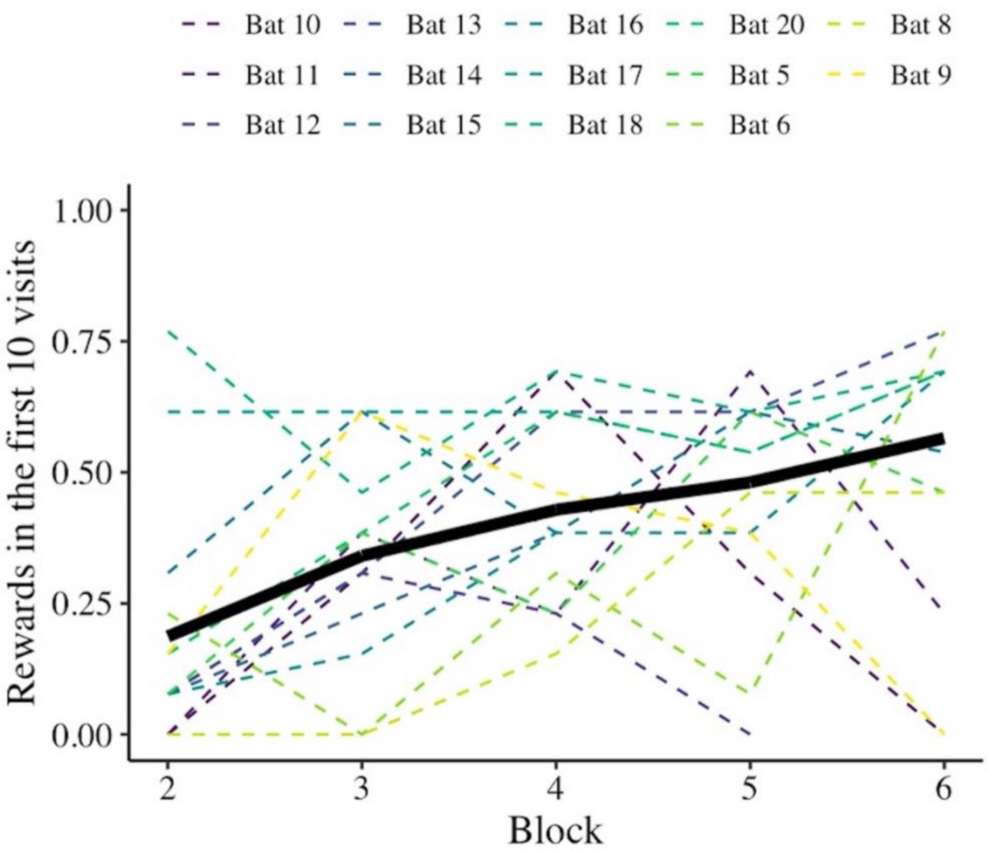

Fig S3 Proportion of visits to the rewarding option out of the first 10 visits after each reversal. Thin lines are individual data, and the thick line is the average proportion of rewarded visits over all bats

The effect of reversal is not driven solely by the effect of the first reversal on the first night

The effect of reversal on the visits to the rewarding flower persisted even when the entire data of the first block after the first reversal were removed from the analysis, and a similar GLMM was fit (Figure S4).

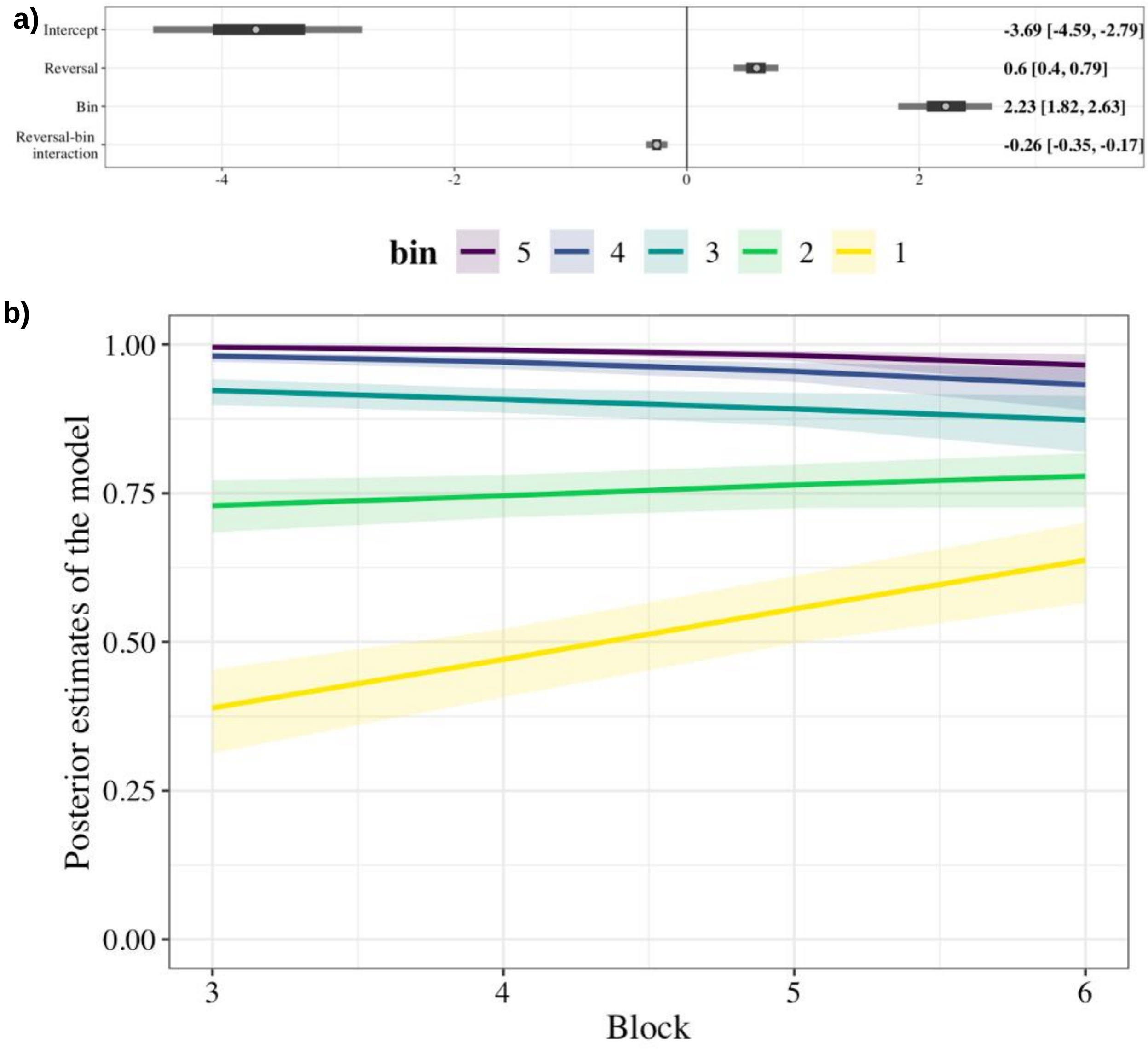

Fig S4 a) Forest plot of the regression coefficients from the model of the effect of reversal and 10-visit bin on the visits to the rewarding flower, excluding the first reversal. Circles represent the means of the posterior distributions of the slope coefficients, thick horizontal lines represent 50% credible intervals, and thin horizontal lines 89% credible intervals. The numbers in bold are the means of the posterior distributions and 89% credible intervals b) Conditional effects plot from the model of the effect of reversal and 10-visit bin on the visits to the rewarding flower - excluding the first reversal - showing the two-way interaction between reversal and bin, sampling from the posterior distribution

In the later stages of the experiment the proportion of rewarded visits did not increase due to reversal experience

At the very start of the second and third nights, in the first bin of visits before any experience of a reversal on that night, the average $Prop_{rew}$ of all the bats was 69.8% [95% CI 64.3, 75], about 7 out of the 10 visits. This was significantly higher than random choice and higher than the $Prop_{rew}$ in the corresponding bin of the first night. Indeed, over all the blocks on these two nights, the bats made significantly more than 50% of their visits to the rewarding flower within 6.67 visits [95% CI 5.42, 7.92] on average. After the first reversal on these nights the $Prop_{rew}$ showed a similar pattern to the first night: a decrease immediately after the reversal and then an increase to near 100%: 94.8% [95% CI 94, 95.6], slightly higher than the 93% [95% CI 91.8, 94.1] $Prop_{rew}$ on the first night.

A GLMM similar to the ones fit to the data from the first night was fit to the data from the second and third nights. Excluding this first block, there was no effect of reversal on the $Prop_{rew}$, although there was a strong effect of 10-visit bin within each block. That is, the proportion of visits to the rewarded flower only increased within each block as the block progressed (Figures S5 and S6).

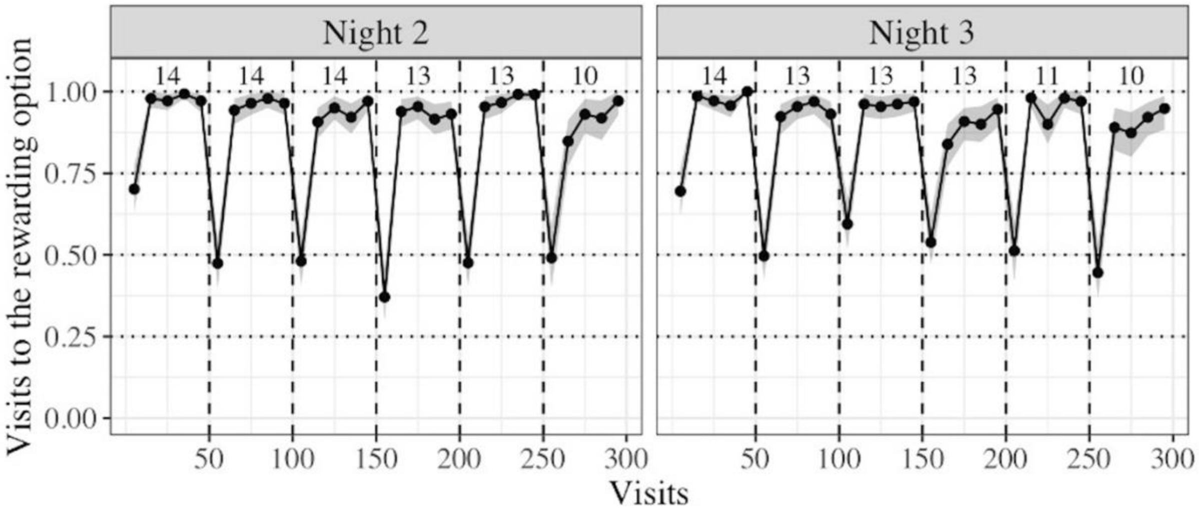

Fig S5 Visits to the rewarding one of two options over second and third experimental nights. Data are average proportions for bins of ten visits averaged over all the individuals that made visits in each bin. Numbers indicate the bats that participated in a block. Shading shows 95% confidence intervals. Dashed lines show reversals

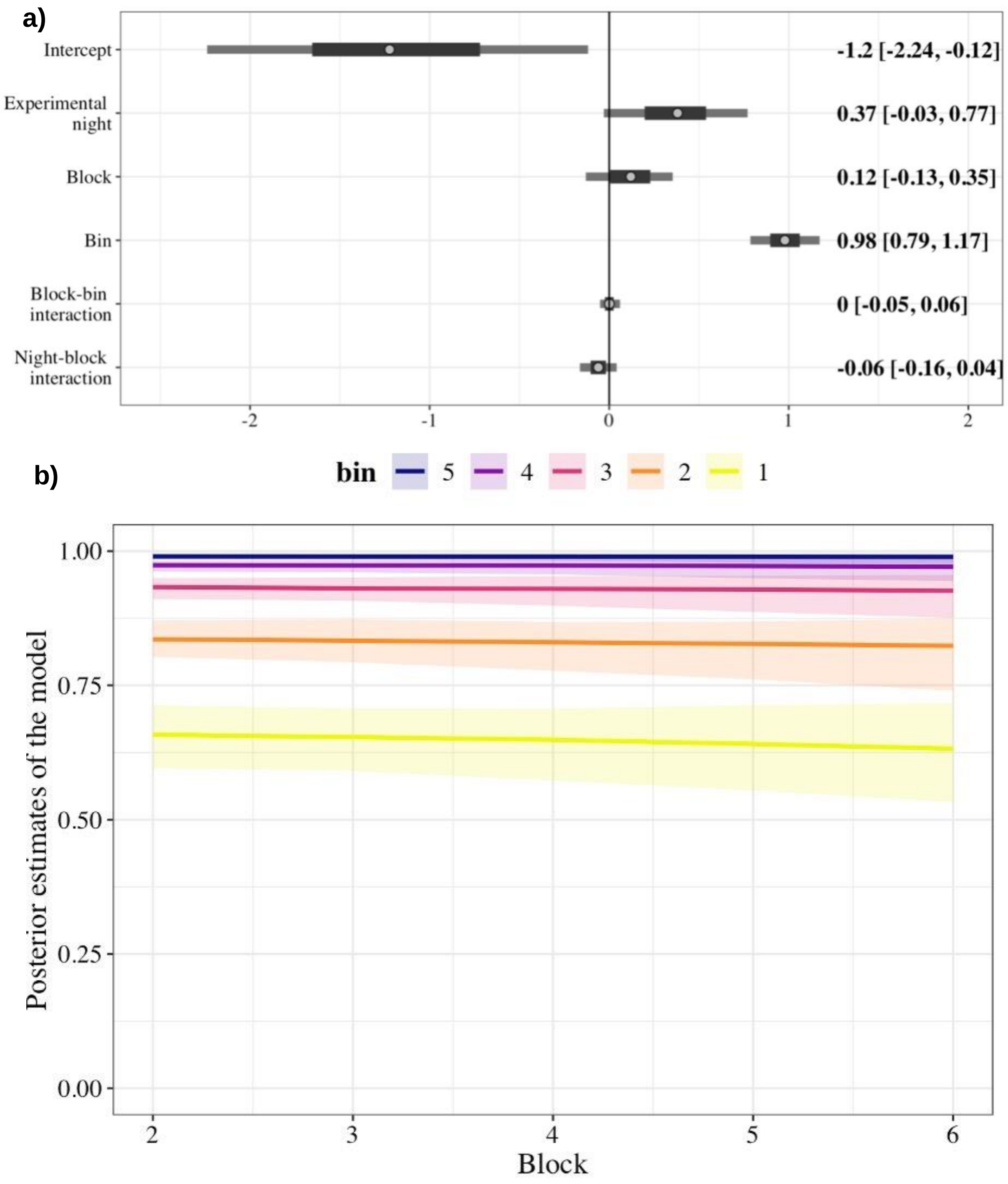

Fig S6 a) Forest plot of the regression coefficients from a model of the effect of experimental night, reversal and 10-visit bin on the visits to the rewarding flower, excluding the first night. Circles represent the means of the posterior distributions of the slope coefficients, thick horizontal lines represent 50% credible intervals, and thin horizontal lines 89% credible intervals. The numbers in bold are the means of the posterior distributions and 89% credible intervals b) Conditional effects plot from the model of the effect of experimental night, reversal and 10-visit bin on the visits to the rewarding flower - excluding the first night - showing the two-way interaction between reversal and bin, sampling from the posterior distribution
